# Crystal structure elucidation of a geminal and vicinal bis­(tri­fluoro­methane­sulfonate) ester

**DOI:** 10.1107/S2053229624005230

**Published:** 2024-06-14

**Authors:** Thomas Pickl, Julian Zuber, Johannes Stephan, Alexander Pöthig

**Affiliations:** a School of Natural Sciences & Catalysis Research Center (CRC), Technische Universität München, Ernst-Otto-Fischer Strasse 1, 85748 Garching, Germany; J-PARC Center, Japan Atomic Energy Agency, Japan

**Keywords:** crystal structure, bis­(tri­fluoro­methane­sulfonate), geminal ditriflate, vicinal ditriflate, sensitive oil

## Abstract

Geminal and vicinal bis­(tri­fluoro­methane­sulfonate) esters are highly reactive alkyl­ene synthons used as potent electrophiles in the macrocyclization of imid­azoles and the transformation of bypyridines to diquat derivatives *via* nucleophilic substitution reactions. Herein we report the crystal structures of methyl­ene (C_3_H_2_F_6_O_6_S_2_) and ethyl­ene bis­(tri­fluoro­methane­sulfonate) (C_4_H_4_F_6_O_6_S_2_), the first examples of a geminal and vicinal bis­(tri­fluoro­methane­sulfonate) ester characterized by single-crystal X-ray diffraction (SC-XRD). With melting points slightly below ambient temperature, both reported bis­(tri­fluoro­methane­sulfonate)s are air- and moisture-sensitive oils and were crys­tallized at 277 K to afford two-com­ponent non-merohedrally twinned crystals. The dominant inter­actions present in both com­pounds are non-classical C—H⋯O hydrogen bonds and inter­molecular C—F⋯F—C inter­actions between tri­fluoro­methyl groups. Mol­ecular electrostatic potential (MEP) cal­culations by DFT-D3 helped to qu­antify the polarity between O⋯H and F⋯F contacts to rationalize the self-sorting of both bis­(tri­fluoro­methane­sulfonate) esters in polar (non-fluorous) and non-polar (fluorous) domains within the crystal structure.

## Abstract 2

The crystal structures of methyl­ene and ethyl­ene bis­(tri­fluoro­methane­sulfonate) are reported, which represent the first crystallographic characterization of a geminal and vicinal bis­(tri­fluoro­methane­sulfonate) ester.

## Introduction

Tri­fluoro­methane­sulfonate (triflate) is an important functional group in organic chemistry owing to its strong electron-withdrawing nature (Howells & McCown, 1977 ▸; Hendrickson *et al.*, 1977 ▸). It is an excellent leaving group used in many organic transformations, such as nucleophilic substitutions, due to the ex­treme stability of the liberated triflate anion (OTf^−^). Thus, the derived triflyl esters (*R*–OTf) are potent electrophiles, re­presenting a halogen-free alternative to alkyl halides in nucleophilic substitution reactions.

Pushing the reactivity of these com­pounds to an extreme, two triflate groups can be attached to the same carbon to form geminal bis­(triflate) esters with the general formula TfO–C*R*_2_–OTf (Martínez *et al.*, 1979 ▸, 1987 ▸). Among other landmark examples, the parent com­pound methyl­ene bis­(triflate) (**1**, *R* = H) had already been reported in 1980 (Katsuhara & DesMarteau, 1980 ▸) but has not been used in chemical synthesis until several decades later. As a highly reactive C1 synthon, the electrophilicity of **1** was eventually harnessed to construct large cyclo­phanes *via* nucleophilic substitution reactions (Anneser *et al.*, 2015 ▸), particularly in cases where bis­(imid­azoles) were macrocyclized to methyl­ene-bridged tetra­(imidazolium) salts (Altmann *et al.*, 2015 ▸, 2016 ▸; Bernd *et al.*, 2020 ▸).

Similarly, the ethyl­ene-bridged bis­(triflate) ester TfO–(CH_2_)_2_–OTf (**2**) has been commonly used as a bis­-alkyl­ating reagent (C2 synthon), among others, for the transformation of bi­pyridines to diquat derivatives (Coe *et al.*, 2006 ▸) and for the synthesis of ethyl­ene-bridged metal com­plexes (Lindner *et al.*, 1990 ▸) or η^2^-olefin metal com­plexes (Lindner *et al.*, 1985 ▸).

Investigating the structure–property relationship of geminal and vicinal bis­(sulfonate) esters, such as com­pounds **1** and **2**, respectively, is imperative to gain a better understanding of their reactivity. In this regard, a structural com­parison of similar alkyl­ene bis­(mesylates) used as DNA cross­linking agents has been reported, which includes the parent com­pound MsO–(CH_2_)_2_–OMs (**3**, Ms = mesyl or methane­sulfon­yl) (McKenna *et al.*, 1989 ▸). So far, this study has been complemented by the structural characterizations of only a few other vicinal bis­(mesylate) and bis­(tosyl­ate) derivatives, such as TsO–(CH_2_)_2_–OTs (**4**, Ts = tosyl or toluene­sulfon­yl) (Groth *et al.*, 1985 ▸), and a handful of geminal bis­(tosyl­ates) (Kamal *et al.*, 2020 ▸).
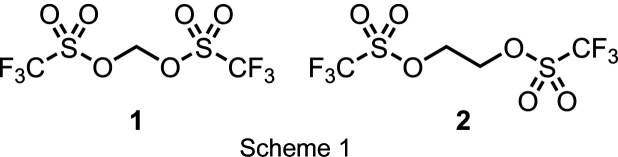


To date, however, there are no reports on the mol­ecular structures of geminal or vicinal bis­(triflate) esters, such as the title com­pounds **1** and **2** (see Scheme 1). Aiming to study the structure–reactivity relationship of these alkyl­ene sources, we synthesized both com­pounds and characterized them in the solid state by single-crystal X-ray diffraction (SC-XRD).

## Experimental

### Synthesis and crystallization

Sulfonate esters **1** and **2** were prepared according to es­tab­lished procedures. Methyl­ene bis­(triflate) (**1**) was synthesized by heating an equimolar suspension of triflic anhydride and paraformaldehyde to 353 K, causing the liberation of formaldehyde, which was further reacted with the anhydride at the same temperature for 16 h. Following the evaporation of excess triflic anhydride *in vacuo*, the crude product was passed over a short plug of silica with di­chloro­methane as the eluent. After removal of all volatiles at 293 K under reduced pressure, analytically pure **1** was obtained as a colourless-to-brown oil. The yields of this reaction typically range between 15 and 20%, which is consistent with previous reports (An­neser *et al.*, 2015 ▸).

There are several approaches for the preparation of ethyl­ene bis­(triflate) (**2**), *e.g.* the straightforward transmetalation of ethyl­ene dibromide with AgOTf (Shackelford *et al.*, 1985 ▸). However, for the purpose of this study, the reaction of ethyl­ene glycol with triflic anhydride under basic conditions was chosen as the preferred method because the diol is readily available and inexpensive, and the desired product is usually obtained in close to qu­anti­tative yields (Kuroboshi *et al.*, 2015 ▸). To equimolar amounts of triflic anhydride and pyridine in di­chloro­methane was added half an equivalent of ethyl­ene glycol at 273 K. The reaction mixture was stirred at the same temperature for 45 min, filtered and washed several times with water. The organic layer was dried over sodium sulfate, filtered and concentrated under reduced pressure. The crude product was filtered over a short plug of silica with di­chloro­methane as the eluent. All volatiles were subsequently re­moved at 293 K under reduced pressure to afford analytically pure **2** as a colourless oil (81% yield).

Bis(triflates) **1** and **2** are air- and water-sensitive liquids at ambient temperature, with melting points between 278 and 288 K (Lindner *et al.*, 1981 ▸; Anneser *et al.*, 2015 ▸). Single crystals were grown by allowing the com­pounds to solidify slowly over the course of several hours at a temperature of 277 K. To prevent the obtained crystals from melting immediately during picking, the tools used in the process were cooled by repeatedly submerging them in a Dewar flask filled with liquid nitro­gen. Additionally, a piece of dry ice was placed on the microscope slide to delay the melting of the specimen on the glass. The selected crystals were then mounted on top of a Kapton micro sample holder (MicroMount) coated with perfluorinated ether and rapidly transferred to the diffractometer.

### Refinement

Data collection and structure refinement details are sum­marized in Table 1 ▸. As implemented in *APEX4* (Bruker, 2022 ▸), the non-merohedral twinning of **1** and **2** was addressed by integration of the diffraction data using two orientation matrices in *SAINT* (Bruker, 2019 ▸), followed by scaling and absorption correction with *TWINABS* (Bruker, 2012 ▸). The structures were solved by *SHELXT* (Sheldrick, 2015*a* ▸) and refined against the respective HKLF5 files using *SHELXL* (Sheldrick, 2015*b* ▸) in conjunction with *ShelXle* (Hübschle *et al.*, 2011 ▸). All non-H atoms were refined with anisotropic displacement parameters. H atoms could be located in difference Fourier maps, but for the refinement were positioned geometrically and refined using a riding model, with C—H = 0.99 Å and *U*_iso_(H) = 1.2*U*_eq_(C).

### DFT calculations

All density functional theory (DFT) calculations were per­formed with the *ORCA* quantum chemistry package (Ver­sion 5.0.4; Neese, 2012 ▸, 2022 ▸) using the PBE0 exchange-correlation functional (Adamo & Barone, 1999 ▸) and the def2-TZVP triple-ξ valence basis set (Weigend & Ahlrichs, 2005 ▸), as implemented in *ORCA*. Tighter than normal convergence criteria for SCF calculations (TightSCF) and geometry optimizations (TightOPT) were employed. Grimme’s atom-pairwise dispersion correction with the Becke–Johnson damping scheme (D3BJ) was applied to account for dispersion inter­actions (Grimme *et al.*, 2010 ▸, 2011 ▸). Geometries were optimized in the gas phase without symmetry constraints. The starting geometries were derived from the SC-XRD structures of **1** and **2**. Frequency analysis at the same level of theory as the geometry optimizations confirmed that the calculations had converged to an energetic minimum. To calculate the mol­ecular electrostatic potentials (MEPs), the total SCF density file obtained after a PBE0/def2-TZVP single-point calculation was first converted to a *Gaussian* cube file using the *orca_plot* module implemented in the *ORCA* package. The MEP was then calculated using the *orca-vpot* module and exported in *Gaussian* cube format. With both cube files in hand, the total SCF density was plotted and the MEP was mapped as a colour onto the isosurface in *Molekel* (Version 4.3; Varetto, 2002 ▸).

## Results and discussion

**Figure 1:**
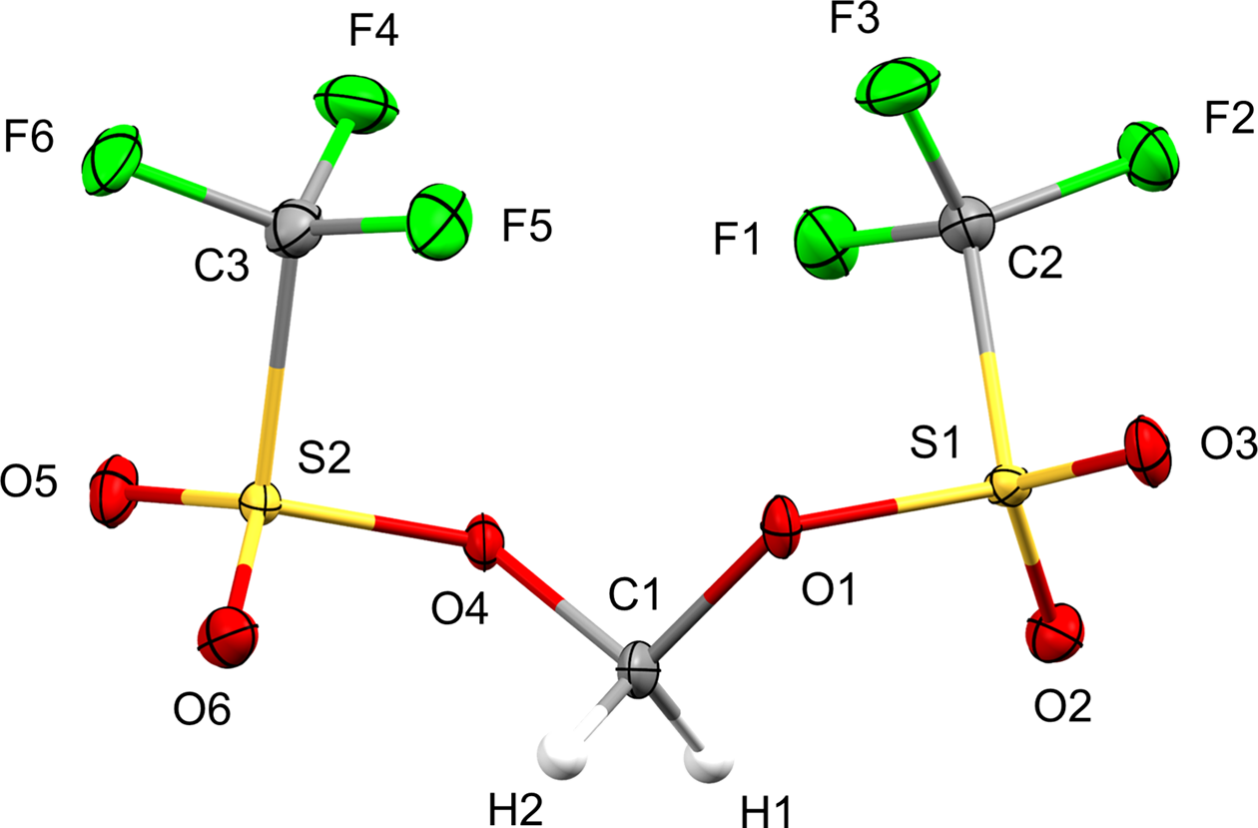
View of methyl­ene bis­(tri­fluoro­methane­sulfonate) (**1**) with the atom-numbering scheme. Displacement ellipsoids for non-H atoms are drawn at the 30% probability level.

**Figure 3:**
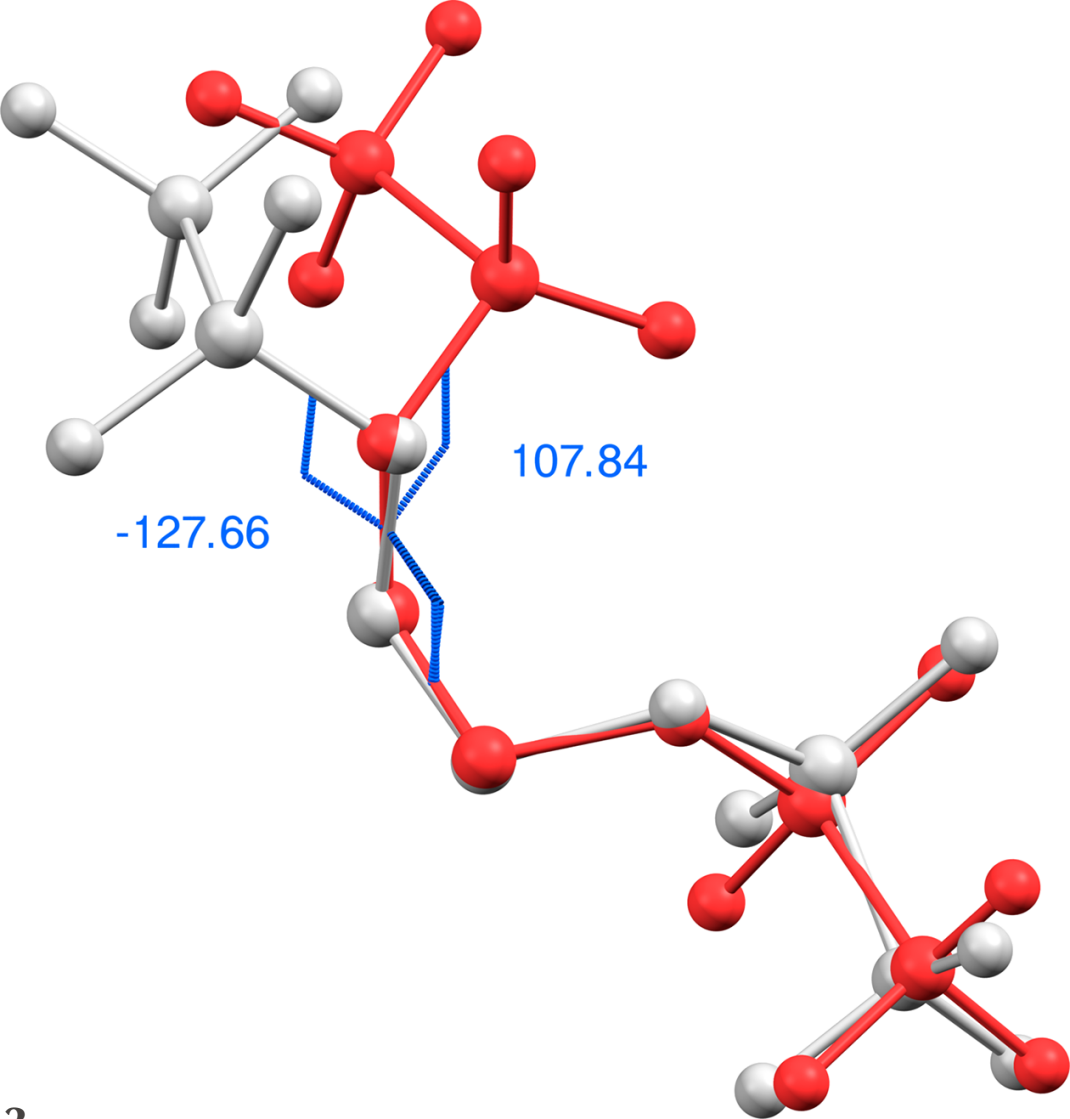
Overlay of the two symmetry-independent conformers of **2**, highlighting the different relative orientations of a tri­fluoro­methane­sulfonate group as qu­anti­fied by different C—C—O—S torsion angles. For clarity, the conformers are drawn in ball-and-stick representation in red and grey, respectively.

Sulfonate esters **1** and **2** both crystallized as two-com­ponent non-merohedral twins, and their asymmetric units contain one and two crystallographically independent mol­ecules, respectively (Figs. 1 ▸ and 2 ▸). Methyl­ene bis­(triflate) (**1**) was found to crystallize in the monoclinic space group *P*2_1_ (No. 4, *Z* = 2), and the fractional contribution of the minor twin com­ponent was refined to 29% in the final model. Ethyl­ene bis­(triflate) (**2**) crystallized in the triclinic space group *P*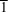 (No. 2, *Z* = 4) with a 37% contribution of the minor twin com­ponent. While both triflic esters are achiral in solution, as indicated by a single ^1^H NMR resonance for the CH_2_ protons (Salomon & Salomon, 1979 ▸; Katsuhara & DesMarteau, 1980 ▸), bis­(triflate) **1** appears to be conformationally locked in the solid state and consequently crystallizes in the Sohncke space group *P*2_1_. Since sulfur is the heaviest atom of the mol­ecule and molybdenum radiation was used in the diffraction experiment, the absolute structure could only be determined with low accuracy. This is reflected by a Flack parameter of 0.12 with a com­parably large standard uncertainty (Flack, 1983 ▸; Parsons *et al.*, 2013 ▸). In contrast to bis­(triflate) **1**, ethyl­ene derivative **2** crystallizes in a centrosymmetric space group (*P*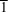) and the asymmetric unit contains two symmetry-independent conformers of the mol­ecule, which differ mainly in the relative orientation of a triflate group, as expressed by different C—C—O—S torsion angles (Fig. 3 ▸). A com­parison of the bond distances of both esters reveals almost identical values for chemically equivalent C—F, C—S and terminal S—O bonds, while the average C—O distance is slightly shorter in **1** (1.434 Å) com­pared to **2** (1.481 Å). In contrast, the mean bond length of the adjacent S—O bond is elongated in **1** (1.573 Å) *versus***2** (1.547 Å).

**Figure 4:**
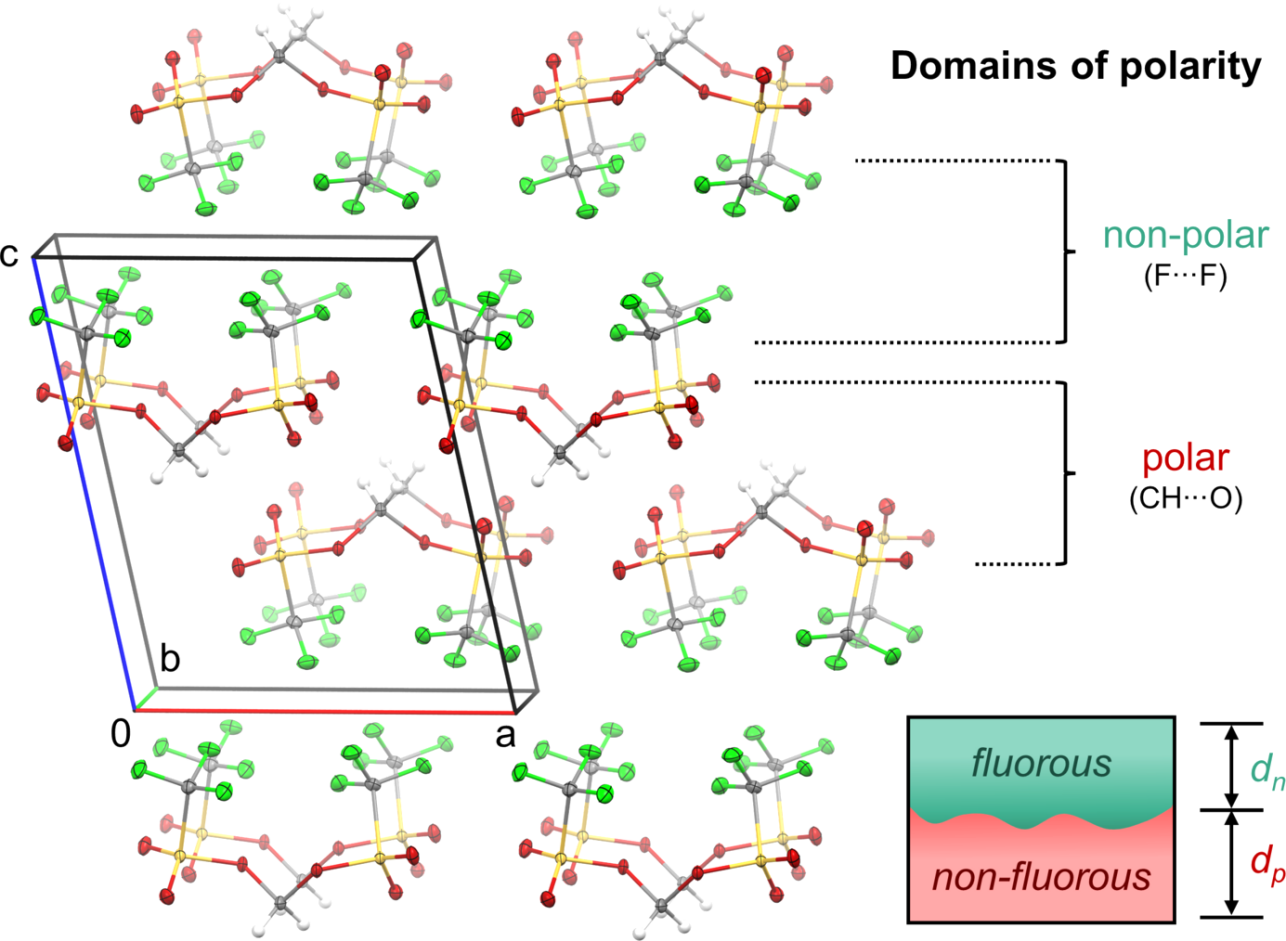
Packing of methyl­ene bis­(tri­fluoro­methane­sulfonate) (**1**), showing alternating two-dimensional layers of fluorous and non-fluorous domains along the *c* axis. The share of these domains of different polarity is indicated by the distances *d*_n_ and *d*_p_, respectively.

**Figure 6:**
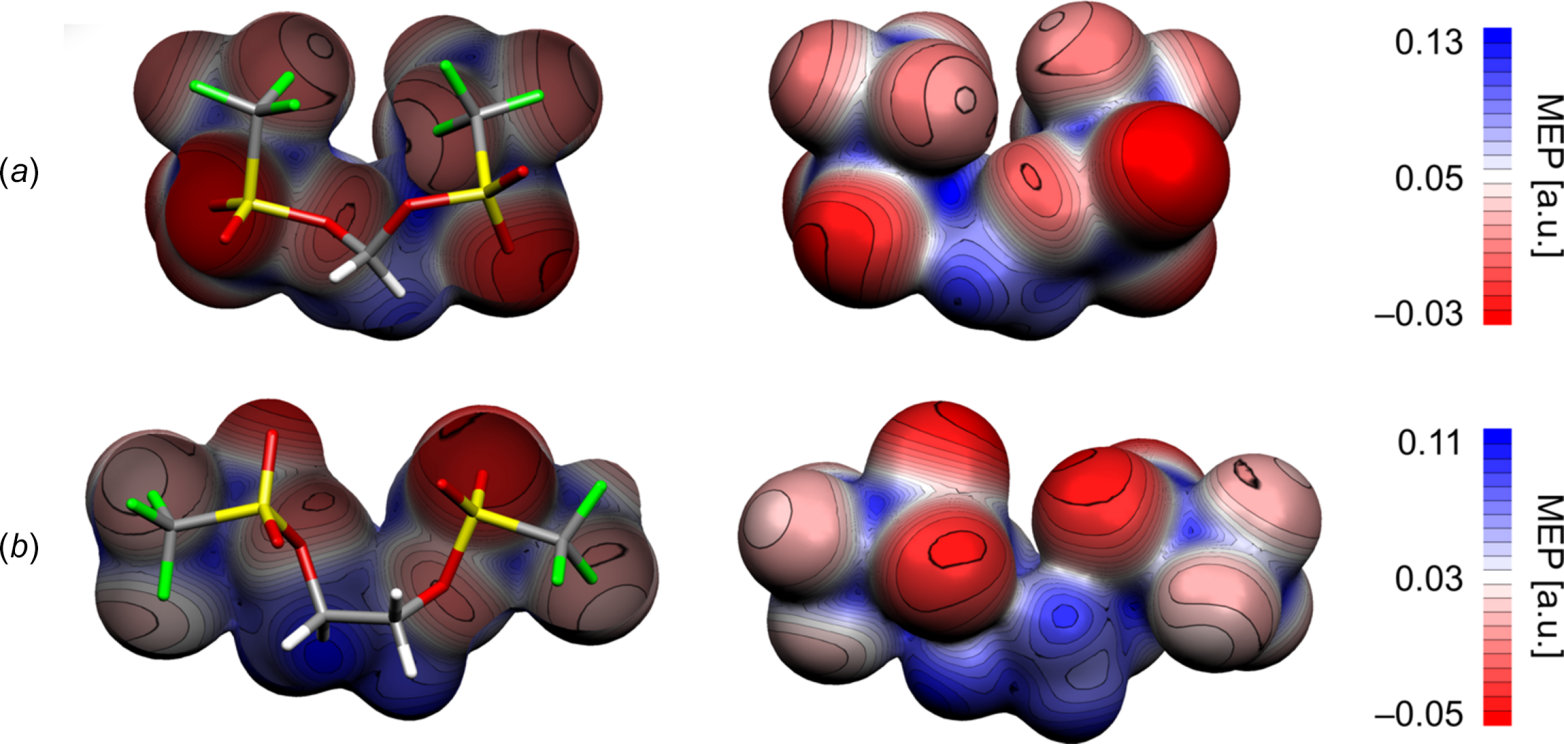
Mol­ecular electrostatic potential (MEP) projected onto the total electron-density surface of (*a*) methyl­ene (**1**) and (*b*) ethyl­ene bis­(tri­fluoro­methane­sulfonate) (**2**). Geometries are optimized by DFT-D3 at the PBE0/def2-TZVP level of theory and MEPs are shown at 0.0062 a.u. electron density.

The packing of bis­(triflates) **1** and **2** is primarily influenced by non-classical C—H⋯O hydrogen bonds between methyl­ene and sulfonate groups, along with inter­molecular C—F⋯F—C inter­actions between tri­fluoro­methyl residues closer than the sum of the van der Waals radii (Haynes, 2015 ▸) (Tables 2 ▸–5 ▸ ▸ ▸). This inter­action pattern results in the formation of two-dimensional fluorous and non-fluorous domains in the crystal packing of **1** and **2** (Figs. 4 ▸ and 5 ▸). Considering this emergence of polar and non-polar domains, we aimed to qu­antify the influence of differently polarized regions within both structures on the overall solid-state arrangement of the bis­(trif­lates). Therefore, we calculated the mol­ecular electrostatic potentials (MEPs) of **1** and **2** based on the optimized geometries of the respective monomers using DFT-D3 in the gas phase (Fig. 6 ▸). As expected, in both cases, the triflate O atoms are the most negatively charged, followed by the F atoms of the CF_3_ groups. In stark contrast, the CH_2_ fragments of methyl­ene and ethyl­ene bis­(triflate) exhibit a high positive charge, which is consistent with their experimentally observed reactivity as strong electrophiles. Along this line, hydrogen-bonding inter­actions occur only between highly charged parts of both bis­(triflates), namely, positively polarized alkyl­ene H and negatively polarized sulfonate O atoms. The tri­fluoro­methyl groups, with a lower C—F bond polarization, do not participate in hydrogen bonding. Instead, they establish fluorous domains whose arrangement in the crystal is governed by the orientation of the CF_3_ groups within the monomers. In methyl­ene bis­(triflate) (**1**), these groups align in a shared direction, whereas in ethyl­ene bis­(triflate) (**2**), they assume opposite orientations in the mol­ecule. The emerging regions of different polarity within the mol­ecules are thus caused by the observed self-sorting of **1** and **2** into highly polar and nonpolar domains within the crystal structure.

To qu­antify the share of these alternating domains within the crystal lattice of **1** and **2**, alternating planes parallel to the *ab* plane were defined by (i) all tri­fluoro­methyl C atoms or (ii) all S atoms of each crystallographically independent molecule contained in the unit cell of both structures. Consequently, the separation of polar and non-polar regions was estimated by calculating the distance between two adjacent planes defined by the S atoms (*d*_p_) or C atoms (*d*_n_) for each crystallo­gra­phi­cally independent molecule (*cf.* Figs. 4 ▸ and 5 ▸). For com­pound **2**, the final *d*_p_ and *d*_n_ values were defined as the average of the individual values of each conformer in the asymmetric unit. For a more detailed definition of the interplanar dis­tances *d*_p_ and *d*_n_, see Fig. S1 in the supporting information. In both structures, the polar region was estimated to be larger than the non-polar (com­pound **1**: *d*_p_ ≃ 3.8 Å, *d*_n_ ≃ 3.5 Å; com­pound **2**: *d*_p_ ≃ 3.7 Å, *d*_n_ ≃ 2.8 Å). Inter­estingly, the determined share of the polar domain in **2** is slightly smaller than in **1**, even though, com­pared to methyl­ene bis­(triflate) (**1**), ethyl­ene congener **2** contains an additional CH_2_ group acting as a hy­drogen-bond donor. Further, the somewhat smaller (polar and non-polar) domain sizes of **2***versus***1** suggest tighter packing of ethyl­ene bis­(triflate) in general. As an overarching trend, this rough estimation of domain size also indicates that roughly the same share can be attributed to the non-fluorous and fluorous regions in both bis­(triflate) structures.

## Conclusion

The first com­prehensive structural analysis of a geminal and vicinal bis­(triflate) ester, specifically methyl­ene (**1**) and ethyl­ene bis­(triflate) (**2**), is presented. Both com­pounds are air- and moisture-sensitive oils under ambient conditions and at low temperature crystallized as non-merohedral two-com­ponent twins. The crystal structures reveal the presence of non-classical C—H⋯O hydrogen bonds and inter­molecular C—F⋯F—C inter­actions, which govern the packing of the com­pounds in the solid state. Mol­ecular electrostatic potential (MEP) calculations of monomers **1** and **2** based on DFT-D3 showed that these inter­actions are driven by the high polarity of the O⋯H contacts and the low polarity of the halogen–halogen contacts, respectively. As a result, bis­(triflates) **1** and **2** self-sort in polar (non-fluorous) and non-polar (fluorous) domains of roughly the same relative size within the crystal lattice.

## Supplementary Material

Crystal structure: contains datablock(s) global, 2, 1. DOI: 10.1107/S2053229624005230/oj3019sup1.cif

Structure factors: contains datablock(s) 1. DOI: 10.1107/S2053229624005230/oj30191sup2.hkl

Supporting information file. DOI: 10.1107/S2053229624005230/oj30191sup4.cdx

Supporting information file. DOI: 10.1107/S2053229624005230/oj30191sup6.cml

Structure factors: contains datablock(s) 2. DOI: 10.1107/S2053229624005230/oj30192sup3.hkl

Supporting information file. DOI: 10.1107/S2053229624005230/oj30192sup5.cdx

Supporting information file. DOI: 10.1107/S2053229624005230/oj30191sup6.cml

Supporting information file. DOI: 10.1107/S2053229624005230/oj30192sup7.cml

Packing of the title compounds. DOI: 10.1107/S2053229624005230/oj3019sup8.pdf

CCDC references: 2360162, 2360161

## Figures and Tables

**Figure 2:**
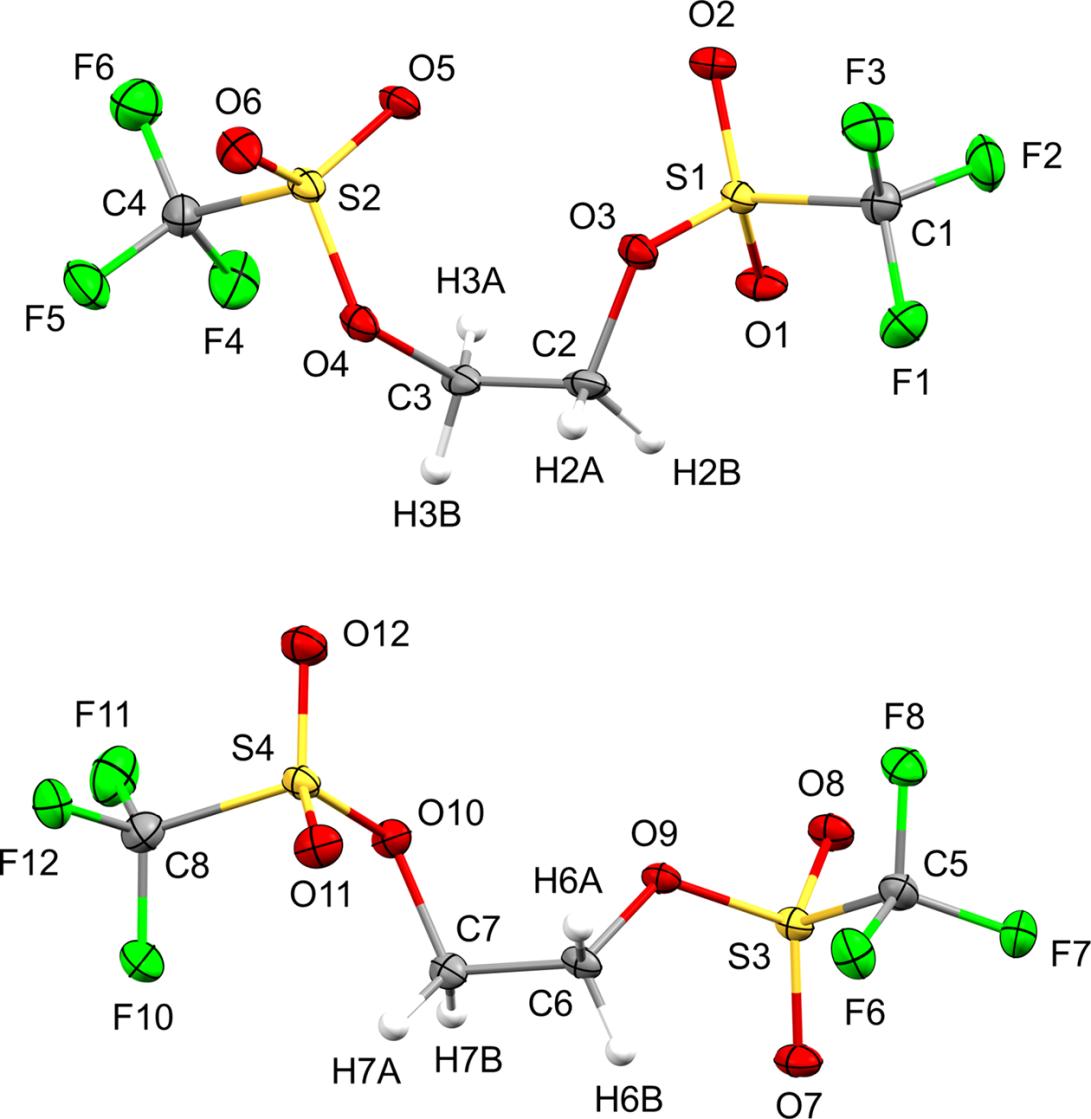
View of both symmetry-independent conformers of ethyl­ene bis­(tri­fluoro­methane­sulfonate) (**2**) with the atom-numbering scheme. Displacement ellipsoids for non-H atoms are drawn at the 30% probability level.

**Figure 5:**
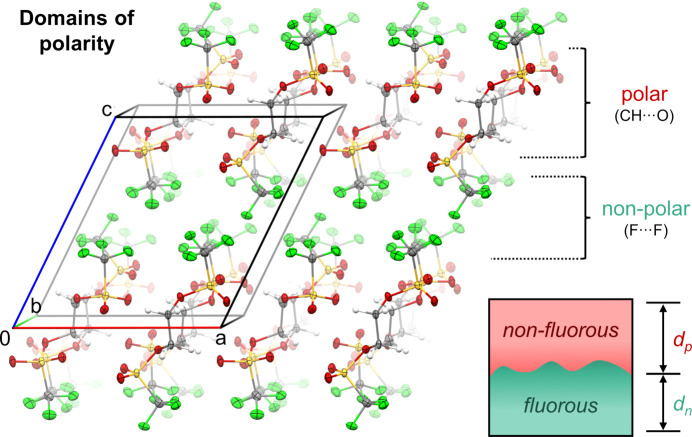
Packing of ethyl­ene bis­(tri­fluoro­methane­sulfonate) (**2**), showing alternating two-dimensional layers of fluorous and non-fluorous domains along the *c* axis. The share of these domains of different polarity is indicated by the distances *d*_n_ and *d*_p_, respectively.

**Table 1:** Experimental details Experiments were carried out at 100 K with Mo *K*α radiation using a Bruker D8 VENTURE diffractometer. Absorption was corrected for by multi-scan methods (*TWINABS*; Bruker, 2012 ▸). H-atom parameters were constrained.

	(**1**)	(**2**)
Crystal data		
Chemical formula	C_3_H_2_F_6_O_6_S_2_	C_4_H_4_F_6_O_6_S_2_
*M*_r_	312.17	326.19
Crystal system, space group	Monoclinic, *P*2_1_	Triclinic, *P* 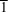
*a*, *b*, *c* (Å)	8.9822 (12), 4.9413 (6), 10.9400 (14)	10.036 (4), 10.664 (3), 11.276 (4)
α, β, γ (°)	90, 102.406 (5), 90	83.540 (9), 64.178 (9), 89.593 (9)
*V* (Å^3^)	474.22 (11)	1078.2 (6)
*Z*	2	4
μ (mm^−1^)	0.68	0.60
Crystal size (mm)	0.35 × 0.32 × 0.06	0.16 × 0.09 × 0.01

Data collection		
*T*_min_, *T*_max_	0.540, 0.746	0.564, 0.745
No. of measured, independent and observed [*I* > 2σ(*I*)] reflections	2295, 2295, 2254	4339, 4339, 3403
*R*_int_	0.044	0.079
(sin θ/λ)_max_ (Å^−1^)	0.667	0.625

Refinement		
*R*[*F*^2^ > 2σ(*F*^2^)], *wR*(*F*^2^), *S*	0.027, 0.072, 1.05	0.066, 0.166, 1.07
No. of reflections	2295	4339
No. of parameters	155	326
No. of restraints	1	0
Δρ_max_, Δρ_min_ (e Å^−3^)	0.41, −0.47	0.60, −0.55
Absolute structure	Flack *x* determined using 959 quotients [(*I*^+^) − (*I*^−^)]/[(*I*^+^) + (*I*^−^)] (Parsons *et al.*, 2013 ▸)	–
Absolute structure parameter	0.12 (4)	–

Computer programs: *APEX4* (Bruker, 2022 ▸), *SAINT* (Bruker, 2019 ▸), *SHELXT* (Sheldrick, 2015*a* ▸), *SHELXL* (Sheldrick, 2015*b* ▸), *ShelXle* (Hübschle *et al.*, 2011 ▸), *PLATON* (Spek, 2020 ▸), *enCIFer* (Allen *et al.*, 2004 ▸) and *FinalCif* (Kratzert, 2023 ▸).

**Table 2:** Hydrogen-bond geometry (Å, °) for **1**[Chem scheme1]

*D*—H⋯*A*	*D*—H	H⋯*A*	*D*⋯*A*	*D*—H⋯*A*
C1—H1⋯O2	0.99	2.32	2.803 (6)	109
C1—H2⋯O6	0.99	2.29	2.802 (6)	111
C1—H1⋯O3^iii^	0.99	2.51	3.416 (5)	152
C1—H2⋯O5^iv^	0.99	2.56	3.430 (5)	147

Symmetry codes: (iii) 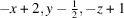; (iv) 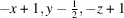.

**Table 3:** Selected interatomic distances (Å) for **1**[Chem scheme1]

F3⋯F6^i^	2.894 (5)	F4⋯F5^i^	2.957 (3)
F1⋯F5^ii^	2.933 (4)		

Symmetry codes: (i) 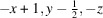; (ii) 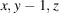.

**Table 4:** Hydrogen-bond geometry (Å, °) for **2**[Chem scheme1]

*D*—H⋯*A*	*D*—H	H⋯*A*	*D*⋯*A*	*D*—H⋯*A*
C2—H2*B*⋯O8^i^	0.99	2.62	3.60 (1)	172
C3—H3*A*⋯O1^iii^	0.99	2.60	2.984 (9)	103
C3—H3*B*⋯O1^iii^	0.99	2.59	2.984 (9)	104
C3—H3*A*⋯O8^iv^	0.99	2.59	3.579 (8)	175
C6—H6*A*⋯O6^v^	0.99	2.34	3.092 (8)	132
C6—H6*A*⋯O11^vi^	0.99	2.47	3.034 (8)	116
C6—H6*B*⋯O2^vii^	0.99	2.45	3.210 (8)	133
C7—H7*A*⋯O5^vii^	0.99	2.49	3.365 (8)	147
C7—H7*B*⋯O7^viii^	0.99	2.70	3.525 (7)	142
C7—H7*B*⋯O2^iv^	0.99	2.54	3.349 (9)	139

Symmetry codes: (i) 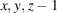; (iii) 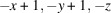; (iv) 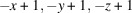; (v) 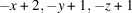; (vi) 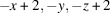; (vii) 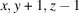; (viii) 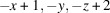.

**Table 5:** Selected interatomic distances (Å) for **2**[Chem scheme1]

F5⋯F7	2.823 (7)	F3⋯F9^ii^	2.954 (5)
F7⋯F12^i^	2.951 (7)		

Symmetry codes: (i) 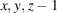; (ii) 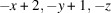.
