# Supplementary material for: Crystal structure elucidation of a geminal and vicinal bis­(tri­fluoro­methane­sulfonate) ester
Source: Acta Crystallogr C Struct Chem. 2024 Jun 14;80(Pt 7):278–83. doi: 10.1107/S2053229624005230 (PMC11225611; doi:10.1107/S2053229624005230)
Supplement: Supplementary file 8 [file c-80-00278-sup8.pdf]

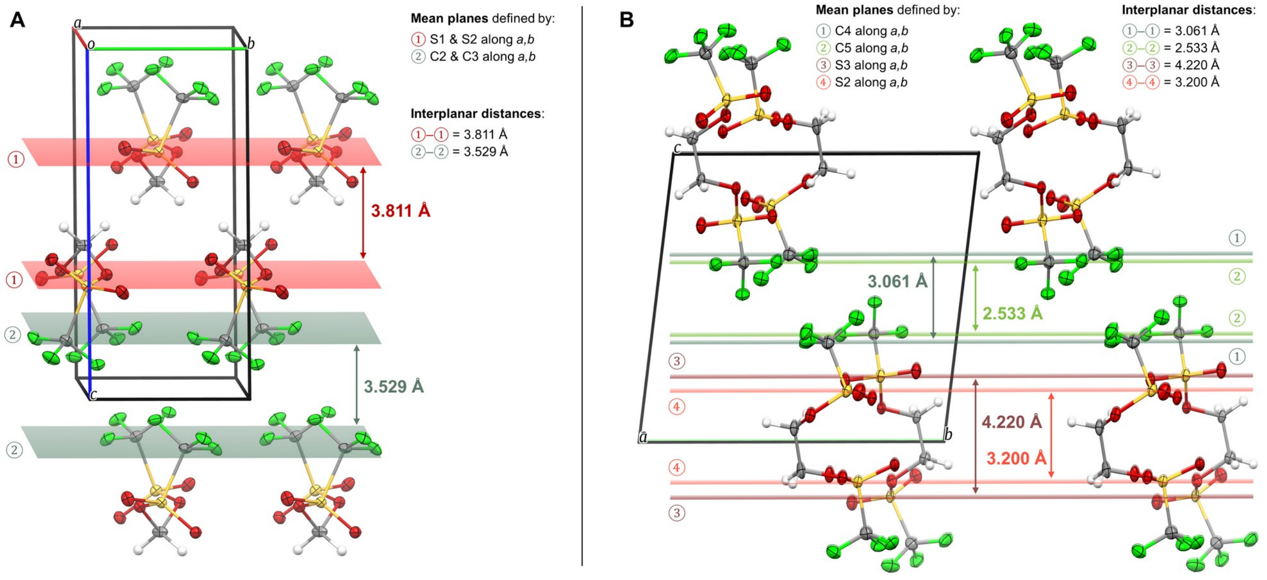

Fig. S1. Packing of methylene (**1**) and ethylene bis(triflate) (**2**) showing mean planes defined by the carbon atoms of CF_3 groups or by sulfur atoms, all of which are parallel to the (001) lattice plane. The distance between two adjacent carbon-containing planes or two adjacent sulfur-containing planes was defined as d_n and d_p, respectively. For compound **2**, two crystallographically independent molecules are found in the asymmetric unit. Accordingly, for each conformer, carbon-planes and sulfur-containing planes were defined in *Mercury* and individual distances (d_n and d_p) were calculated. For comparison with compound **1**, the average of these values was determined.
